# MicroRNA-454 inhibits the malignant biological behaviours of gastric cancer cells by directly targeting mitogen-activated protein kinase 1

**DOI:** 10.3892/or.2022.8276

**Published:** 2022-01-31

**Authors:** Xiaodong Wang, Baichun Liu, Fuxing Wen, Ying Song

Oncol Rep 39: 1494-1504, 2018; DOI: 10.3892/or.2017.6171

Following the publication of this paper, the authors have been unable to obtain consistent results after having repeated some of the flow cytometric assay experiments, undermining their confidence in the reported conclusions concerning the regulatory action of miR-454 on gastric cancer cell apoptosis. Consequently, owing to a lack of confidence in the presented data, the authors have requested that this paper be retracted from the journal.

All authors agree with the retraction of this article, and apologize to the Editor and readership for the inconvenience caused.

